# Supplementary material for: Distribution patterns of fungal community diversity in the dominant tree species Dacrydium pectinatum and Vatica mangachapoi in tropical rainforests
Source: Microbiol Spectr. 2025 Apr 17;13(6):e03092-24. doi: 10.1128/spectrum.03092-24 (PMC12131778; doi:10.1128/spectrum.03092-24)
Supplement: Supplemental figures and table — Figures S1 to S6; Tables S1 to S5. [file spectrum.03092-24-s0001.docx]

**Distribution patterns of fungal community diversity in the dominant tree species *Dacrydium pectinatum* and *Vatica mangachapoi* in tropical rainforests**

Kepeng Ji ^1,2†^, Yaqing Wei^1,3†^, Xin Wang^1,4^, Yu Liu^5^, Rui Sun ^1,3^, Yuwu Li^6*^, Guoyu Lan^1,3*^

The following supporting information is available for this article:

**Supplementary methods**

**Method S1** Detailed description of physicochemical analysis.

Leaf physicochemical properties: Water content (WC) was measured gravimetrically. Organic matter (OM) was measured using the potassium dichromate oxidation method. Total nitrogen (TN) was determined using micro-Kjeldahl digestion followed by steam distillation. Total phosphorus (TP) and total potassium (TK) were assessed using NaOH digestion. Leaf pH was measured in a leaf/water suspension (1:2.5, w/w) using a pH meter (1).

Soil physicochemical properties: Soil pH was determined by taking samples with a soil-to-water ratio of 1:2.5 and measuring it precisely using a pH meter. Soil organic matter (SOM) was determined using the potassium dichromate heating method. Soil water content (WC) was measured gravimetrically. Total potassium (TK) was determined using flame photometry. Total phosphorus (TP) was measured using the molybdenum antimony anti-colorimetric method, and total nitrogen (TN) was determined using the indophenol blue colorimetric method. Nitrate nitrogen (NN) and ammonium nitrogen (AN) were determined by steam distillation and indophenol-blue colorimetry, respectively. Available phosphorus (AP) was measured by molybdate-blue colorimetry. Soil samples were first extracted with ammonium acetate before loading the extracts onto an atomic absorption spectrometer with ascorbic acid as a reductant to measure available potassium (AK) (2).

**Method S2** Detailed description of DNA extraction process.

To perform the DNA extraction process, begin by combining 0.5 g of sample with 978 µL of Sodium Phosphate Buffer and 122 µL of MT Buffer in a Lysing Matrix E tube. Vortex the mixture for 40 seconds at a speed of 6 m/s using an MP homogenizer, followed by centrifugation at 14,000 rpm for 10 minutes. Transfer the supernatant to a 1.5 mL centrifuge tube, add 250 µL of PPS, and mix thoroughly before centrifuging again at room temperature at 14,000 rpm for 5 minutes. Next, transfer the supernatant to a 2 mL tube containing 900 µL of Binding Matrix, mix well, and invert for 3 minutes. After a brief spin for 5 seconds, carefully decant the supernatant. Then, add 500 µL of 5.5 M guanidine thiocyanate solution, mix well, and transfer the mixture to a SPINTM Filter. Add 500 µL of SEWS-M and centrifuge at 14,000 rpm for 1 minute, discard the filtrate, and repeat the wash. Discard the liquid in the collection tube and centrifuge at 14,000 rpm for 3 minutes to remove any residual solution, then air-dry for 3 minutes. Finally, add 100 µL of pre-warmed DES elution buffer at 55°C, let it stand for 5 minutes, centrifuge at room temperature at 14,000 rpm for 2 minutes, discard the SPINTM Filter, and collect the total DNA.

**Supplementary figures**

**Figure S1** Study sites on Hainan Island. Red and blue solid circles each represent *V. mangachapoi* and *D. pectinatum* on the map.

**Figure S2** Sampling design on a plot.

**Figure S3** Fungal community composition at class level of *V. mangachapoi and D. pectinatum* in the different compartments.

**Figure S4** Shared and Unique Fungal Genera and Species in the different compartments.

**Figure S5** Fungal sources of different compartments of *D. pectinatum and V. mangachapoi.*

**Figure S6** The relative importance of different ecological processes in dominant fungal class*.*

**Supplementary tables**

**Table S1** Leaves and soil physicochemical properties of *D. pectinatum and V. mangachapoi* in different geographical locations.

**Table S2** Leaves and soil physicochemical properties for different tree species.

**Table S3** Multivariate analysis of variance results on the effects of geographical locations, plant compartments and plant identity on α-diversity (OTU richness) of fungi in *D. pectinatum and* *V. mangachapoi*.

**Table S4** Permuted multivariate analysis of variance (PERMANOVA) tables for differences in fungal community compositions (OTU level).

**Table S5** The classification distribution results of OTUs at different classification levels.

**
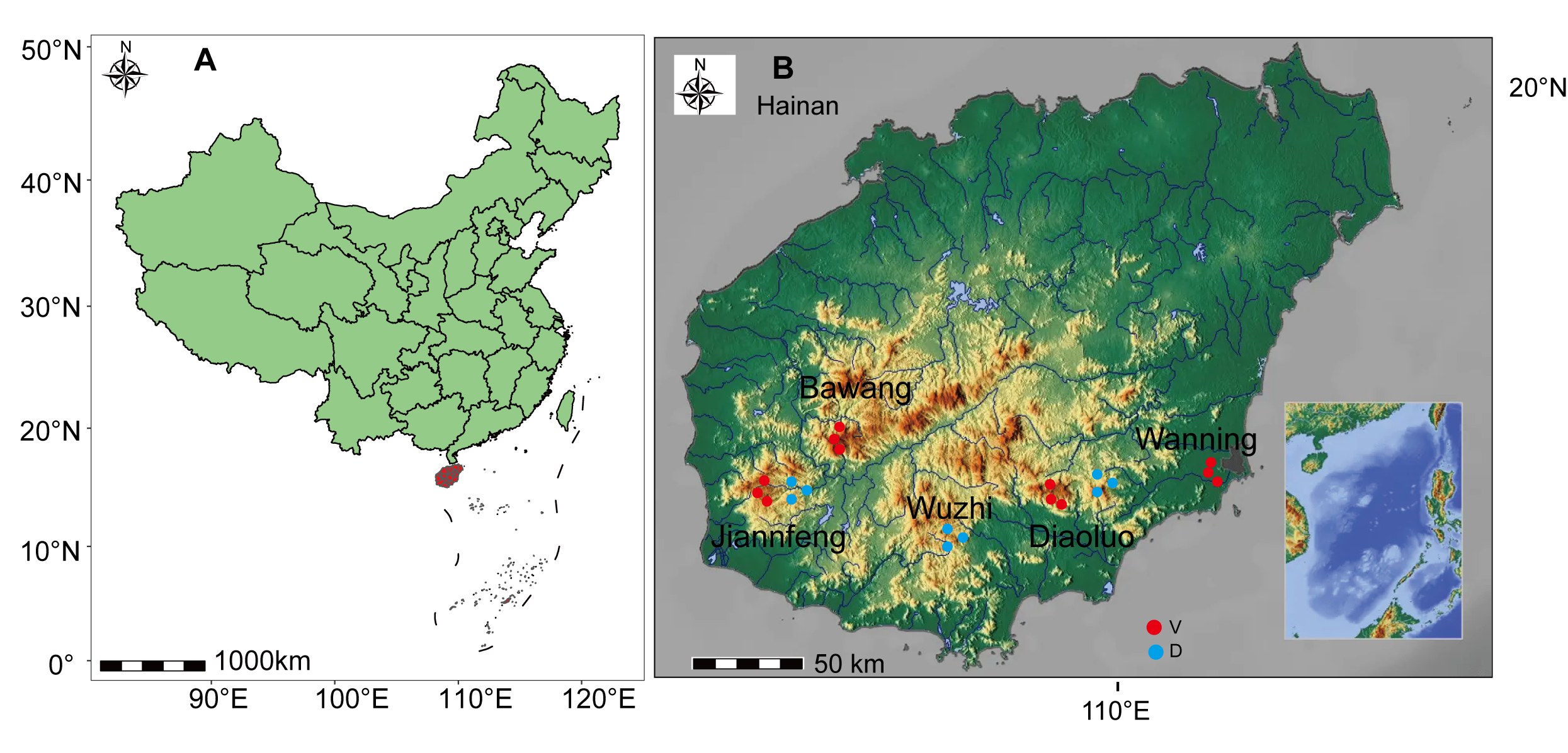
**

**Figure S1** Study sites on Hainan Island. Red and blue solid circles each represent *V. mangachapoi* and *D. pectinatum* on the map.


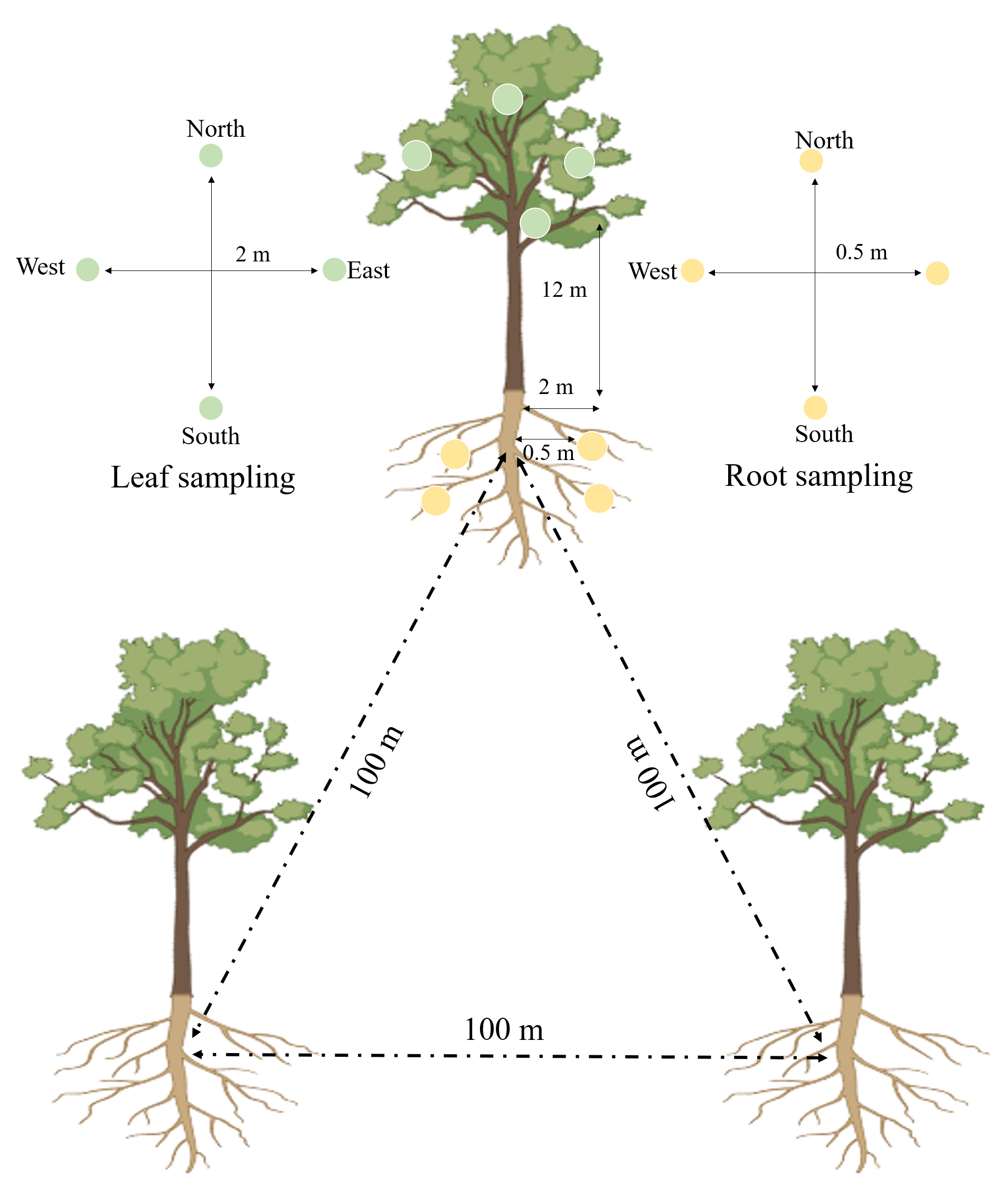


**Figure S2** Sampling design on a plot. For each plot, we selected three trees for sampled root (yellow solid circles) and leaves (green solid circles) from all four cardinal directions. Then three trees of a plot were mixed to form a single composite sample.


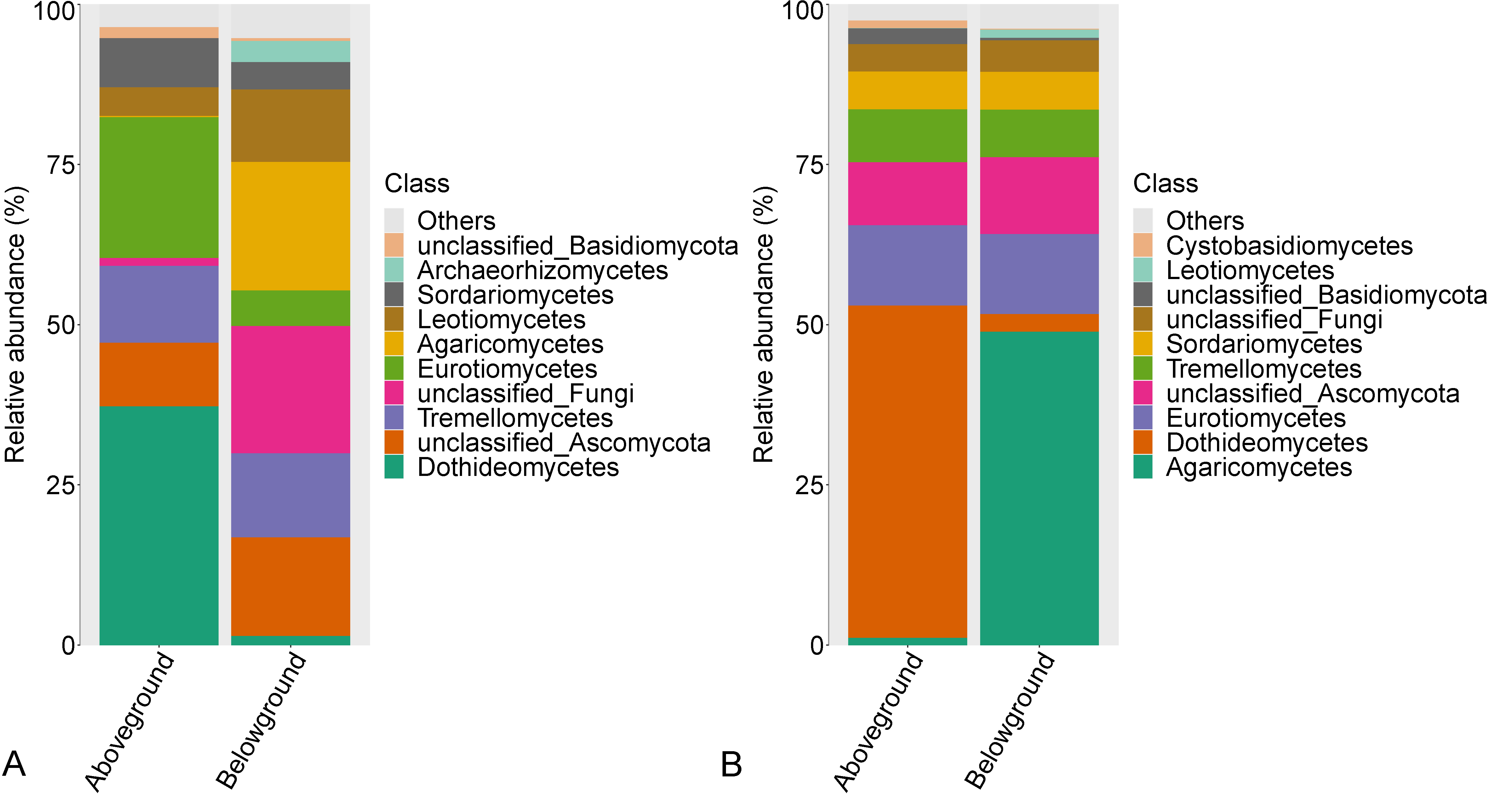


**Figure S3** Fungal community composition at class level of *V. mangachapoi and D. pectinatum* in the different compartments. A: *D. pectinatum*; B: *V. mangachapoi*.


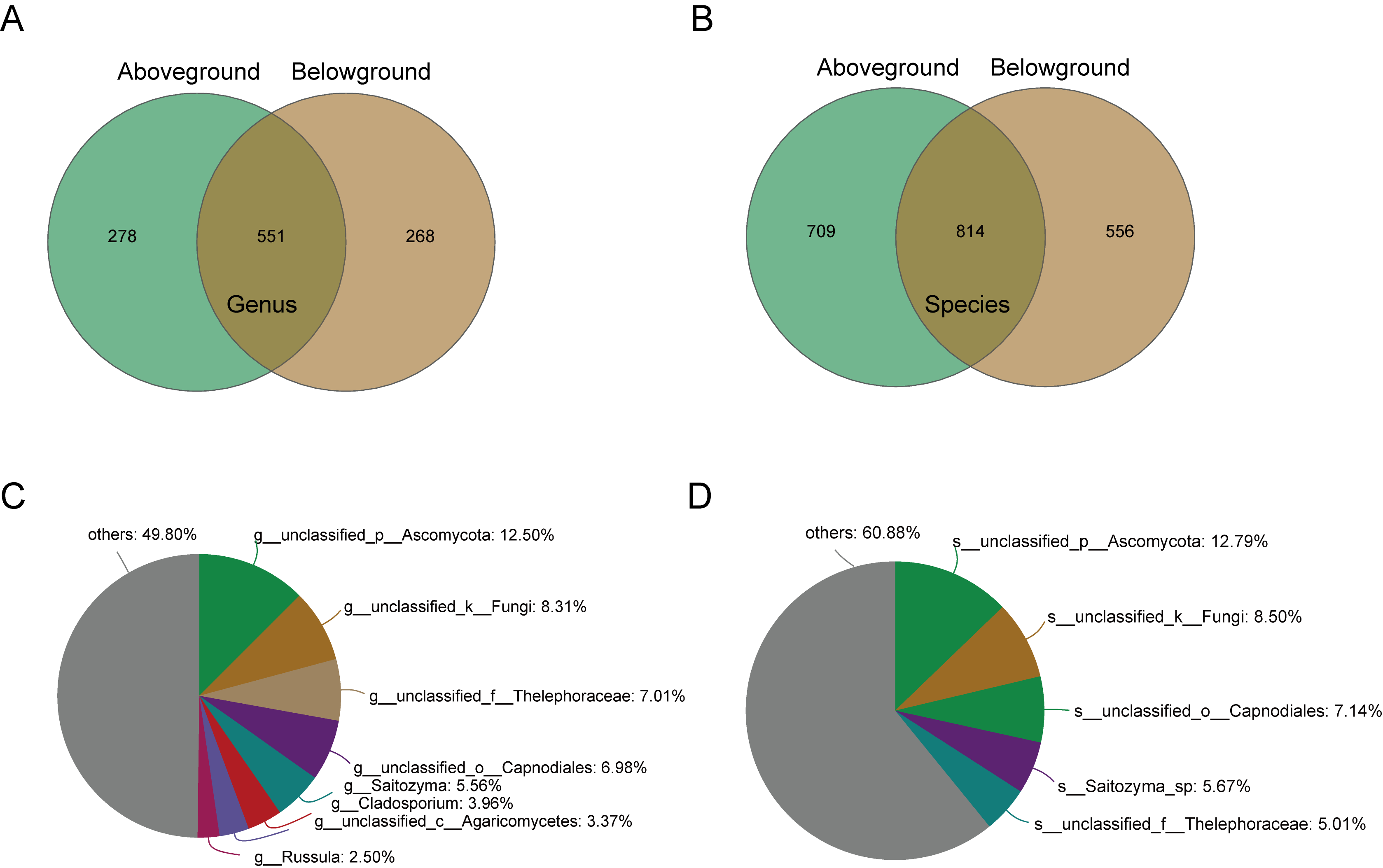


**Figure S4** Shared and Unique Fungal Genera and Species in the different compartments. A and B: The numbers of shared and unique fungal genera and species; C: Relative abundance of shared fungal genera; D: Relative abundance of shared fungal species.

**
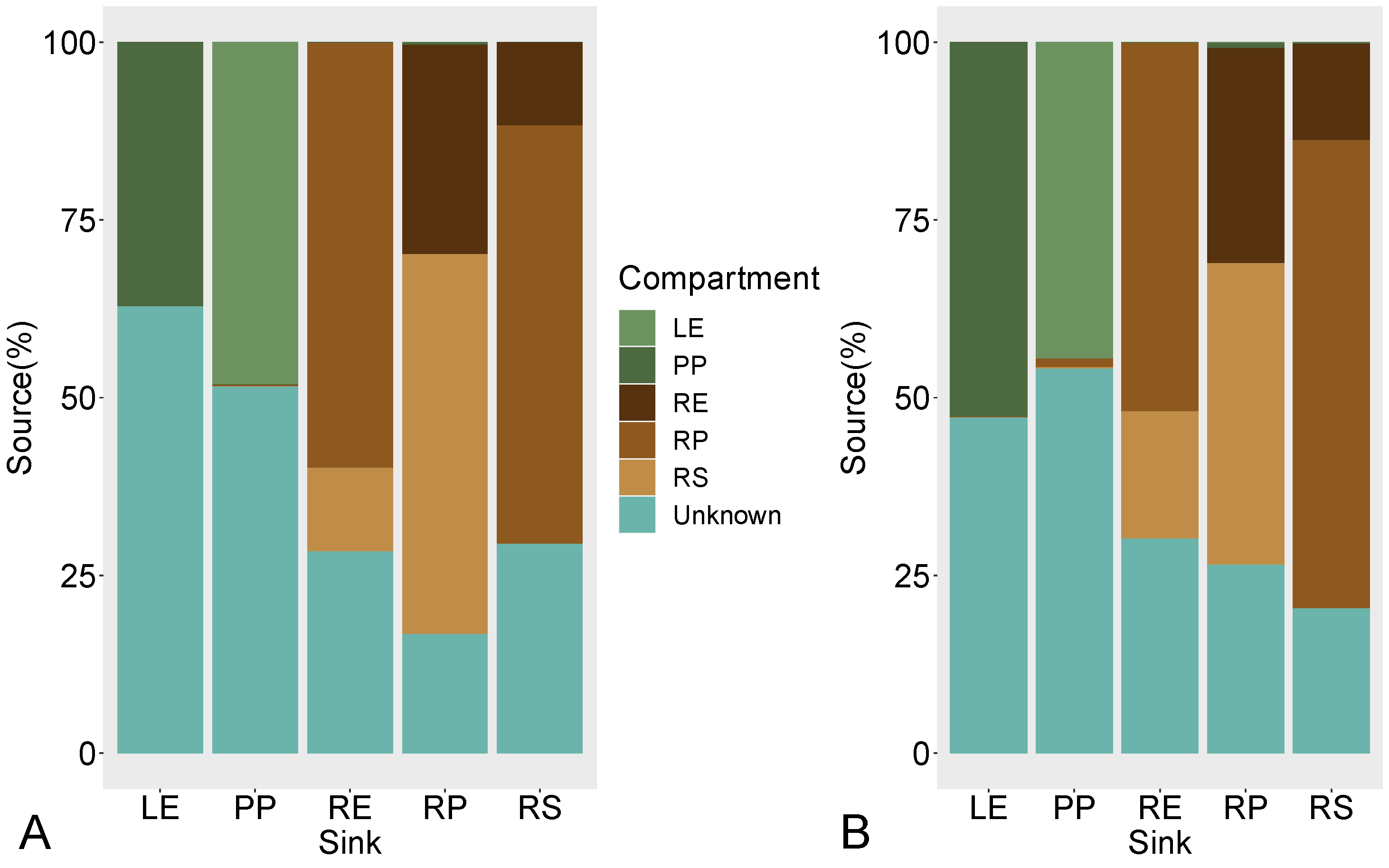
**

**Figure S5** Fungal sources of different compartments of *D. pectinatum* and *V. mangachapoi.* A: *D. pectinatum*; B: *V. mangachapoi*. Compartment abbreviation: LE, Leaf endophytic; PP, Leaf epiphytic; RP, Rhizoplane; RS, Rhizosphere; RE, Root endosphere.


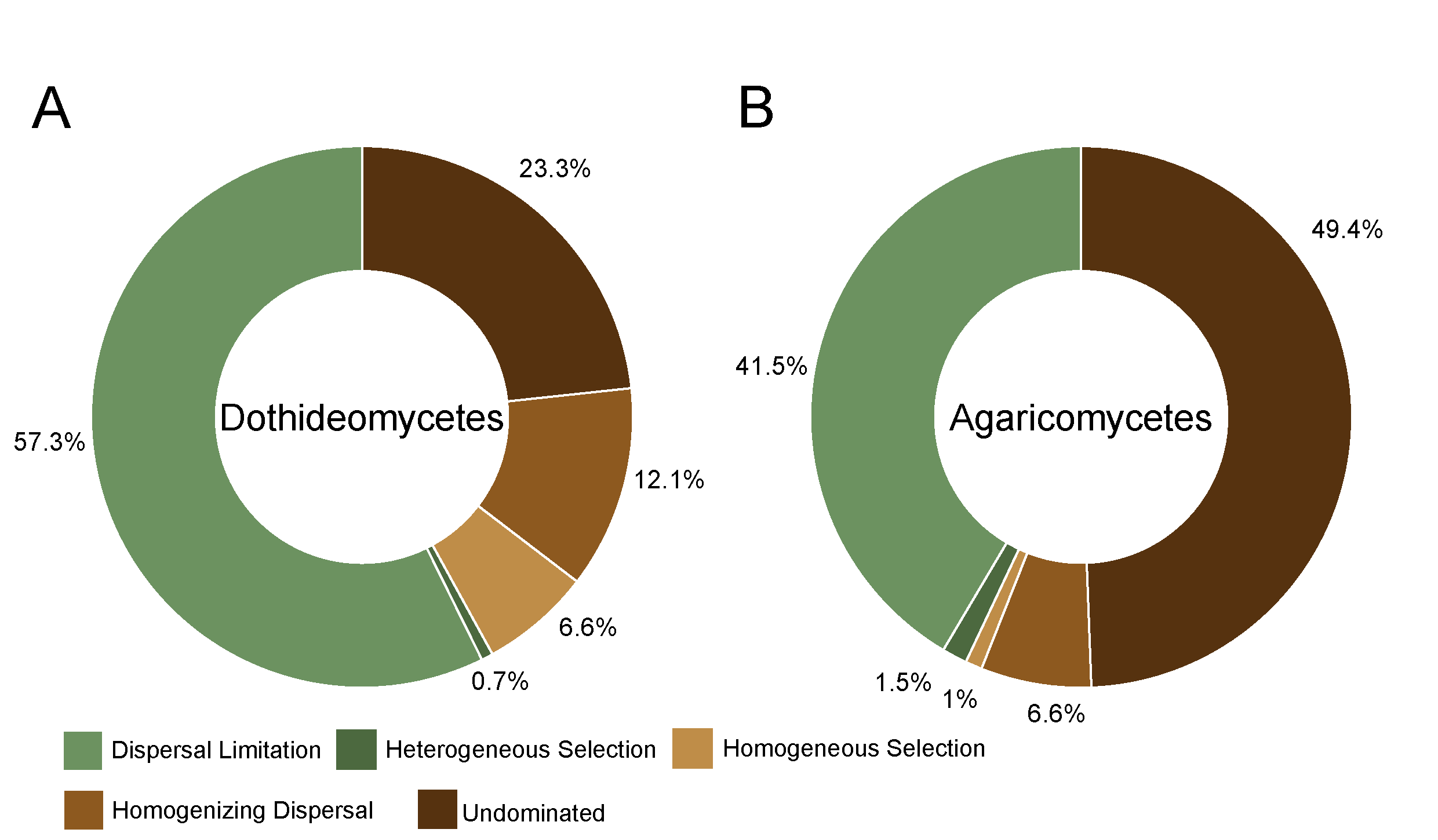


**Figure S6** The relative importance of different ecological processes in dominant fungal class*.*

**Table S1** Leaves and soil physicochemical properties of *D. pectinatum and V. mangachapoi* in different geographical locations.

| **Environmental variables** | **Compartment** | | **BW** | **DL** | **WZ** | **JF** | **WN** |
| --- | --- | --- | --- | --- | --- | --- | --- |
| Water contents (%) | | Leaf | 52.58±1.41 | 55.44±3.27 | 58.82±1.85 | 52.25±3.30 | 62.01±5.75 |
|  | | Soil | 18.22±1.94 | 12.53±9.15 | 39.05±11.57 | 12.20±11.34 | 2.32 ±0.13 |
| Organic matter (%) | | Leaf | 43.69±1.26 | 45.54±1.58 | 50.30±0.95 | 49.28±0.12 | 46.23±1.44 |
|  | | Soil | 4.09±0.68 | 4.14±2.12 | 20.42±6.76 | 4.65±1.48 | 1.66±0.12 |
| pH | | Leaf  Soil | 4.85±0.15  5.01±0.12 | 4.35±0.30  5.59±1.05 | 4.10±0.16  4.54±0.14 | 4.64±0.15  4.79±0.36 | 4.57±0.16  5.25±0.21 |
| Total nitrogen (%) | | Leaf  Soil | 3.53±0.14  0.22±0.02 | 2.95±0.40  0.13±0.06 | 1.52±0.64  0.43±0.10 | 2.58±0.43  0.16±0.06 | 4.68±0.79  0.06±0.01 |
| Total phosphorus(g/kg) | | Leaf  Soil | 0.24±0.01  0.07±0.01 | 0.22±0.07  0.04±0.01 | 0.13±0.02  0.08±0.02 | 0.16±0.02  0.03±0.01 | 0.44±0.11  0.03±0.01 |
| Total potassium (g/kg) | | Leaf  Soil | 1.26±0.06  2.14±0.13 | 1.27±0.30  2.74±0.76 | 1.14±0.16  1.33±0.23 | 1.33±0.29  1.35±1.21 | 1.96±0.68  0.76±0.03 |
| Magnesium (coml/kg) | | Leaf | 0.19±0.01 | 0.13±0.03 | 0.09±0.02 | 0.11±0.02 | 0.18±0.01 |
| Calcium (coml/kg) | | Leaf | 0.74±0.04 | 0.61±0.31 | 0.48±0.08 | 0.31±0.05 | 0.46±0.11 |
| Available potassium (mg/kg) | | Soil | 98.97±6.00 | 67.48±19.41 | 89.91±3.54 | 80.01±30.70 | 17.00±1.31 |
| Available phosphorus (mg/kg) | | Soil | 5.58±1.04 | 3.00±1.09 | 4.80±1.29 | 2.39±0.77 | 1.42±0.04 |
| Nitrate nitrogen (mg/kg) | | Soil | 17.53±3.61 | 7.64±3.32 | 13.29±0.97 | 10.93±3.71 | 6.52±1.78 |
| Ammonium nitrogen (mg/kg) | | Soil | 4.29±0.07 | 4.97±1.28 | 11.56±0.65 | 7.16±2.03 | 4.20±0.87 |

**Table S2** Leaves and soil physicochemical properties in different tree species. Different lowercase letters in the same row indicated significant differences (*P* < 0.05).

| **Environmental variables** | **Compartment** | ***V. mangachapoi*** | ***D. pectinatum*** |
| --- | --- | --- | --- |
| WC (%)  SOM (%)  pH  TN (%)  TP (%)  TK (%)  Ca (coml/kg)  Mg (coml/kg)  WC (%)  SOM (%)  pH  TN (%)  TP (%)  TK (%)  AN (mg/kg)  NN (mg/kg)  AP (mg/kg)  AK (mg/kg)  Rainfall(mm)  Temperature (℃) | Leaf  Leaf  Leaf  Leaf  Leaf  Leaf  Leaf  Leaf  Soil  Soil  Soil  Soil  Soil  Soil  Soil  Soil  Soil  Soil | 54.54±5.97a  45.89±2.53a  4.63±0.26a  3.60±0.83a  0.28±0.13a  1.51±0.50a  0.50±0.19a  0.16±0.03a  6.51±7.22a  3.03±1.40a  5.44±0.74a  0.12±0.07a  0.04±0.02a  1.67±0.95a  4.47±1.13a  10.00±5.77a  3.35±1.85a  66.17±37.72a  122.18±99.14a  24.45±1.50a | 56.88±3.04a  48.77±1.83b  4.32±0.31b  2.14±0.68b  0.15±0.03b  1.17±0.23a  0.52±0.30a  0.10±0.02b  27.67±11.91b  10.55±8.58b  4.6±0.36b  0.27±0.14b  0.05±0.03a  1.91±1.19a  8.81±2.52b  11.49±3.37a  3.06±1.55b  78.73±23.25b  53.36±32.71a  23.27±2.12a |

**Table S3** Multivariate analysis of variance results on the effects of geographical locations, plant compartments and plant identity on α-diversity (OTU richness) of fungi in *D. pectinatum* and *V. mangachapoi*.

| **Dataset** | **Variables** | **Pseudo F** | ***p*-value** |
| --- | --- | --- | --- |
| ALL | Compartment | 108.240 | <0.001 |
|  | Species | 0.578 | 0.449 |
|  | Location | 8.655 | <0.001 |
| Phyllosphere | Species | 0.075 | 0.787 |
| Leaf.endosphere | Species | 0.975 | 0.339 |
| Root.endosphere | Species | 3.253 | 0.092 |
| Rhizoplane | Species | 0.809 | 0.383 |
| Rhizosphere | Species | 4.540 | 0.051 |
| Phyllosphere | Location | 11.179 | <0.001 |
| Leaf.endosphere | Location | 5.335 | <0.01 |
| Root.endosphere | Location | 1.515 | 0.168 |
| Rhizoplane | Location | 1.604 | 0.228 |
| Rhizosphere | Location | 3.447 | <0.05 |
|  |  |  |  |

**Table S4** Permuted multivariate analysis of variance (PERMANOVA) tables for differences in fungal community compositions (OTU level).

| Factors | F | *R*^2^ | *P* |
| --- | --- | --- | --- |
| **All**  Location | 3.924 | 0.102 | 0.001 |
| Compartment  Species  Location×Compartment  Location×Species  Compartment×Species  Residual  **LE**  Location  Species  Location×Species  Residual  **PP**  Location  Species  Location×Species  Residual  **RE**  Location  Species  Location×Species  Residual  **RP**  Location  Species  Location×Species  Residual  **RS**  Location  Species  Location×Species  Residual | 4.791  4.449  1.615  3.541  1.919  2.659  2.937  2.241  4.957  7.518  4.656  1.469  1.933  1.716  1.548  1.267  1.222  1.608  1.582  1.536 | 0.126  0.029  0.169  0.023  0.050  0.458  0.357  0.098  0.075  0.470  0.431  0.163  0.101  0.304  0.250  0.082  0.073  0.595  0.273  0.056  0.054  0.617  0.273  0.067  0.065  0.594 | 0.001  0.001  0.001  0.001  0.001  0.001  0.001  0.001  0.001  0.001  0.001  0.001  0.001  0.001  0.001  0.125  0.137  0.001  0.006  0.013 |

**Table S5** The classification distribution results of OTUs at different classification levels. Only show the categories that account for more than 2% of all OTUs.

| **Name** | **Taxonomic levels** | **Number of OTUs** | **Percent** |
| --- | --- | --- | --- |
| Ascomycota | Phylum | 9541 | 63.1% |
| unclassified_k__Fungi | Phylum | 2582 | 17.1% |
| Basidiomycota | Phylum | 2471 | 16.3% |
| Dothideomycetes | Class | 2969 | 19.7% |
| c__unclassified_k__Fungi | Class | 5949 | 39.3% |
| Sordariomycetes | Class | 2029 | 13.4% |
| Agaricomycetes | Class | 1271 | 8.4% |
| Eurotiomycetes | Class | 1089 | 7.2% |
| Tremellomycetes | Class | 495 | 3.3% |
| Leotiomycetes | Class | 332 | 2.2% |
| o__unclassified_p__Ascomycota | Order | 2736 | 18.1% |
| o__unclassified_k__Fungi | Order | 2591 | 17.2% |
| o__Capnodiales | Order | 2137 | 14.2% |
| o__Hypocreales | Order | 708 | 4.7% |
| o__Chaetothyriales | Order | 695 | 4.6% |
| o__Xylariales | Order | 509 | 3.4% |
| o__unclassified_c__Sordariomycetes | Order | 490 | 3.2% |
| o__Pleosporales | Order | 451 | 3.0% |
| o__Tremellales | Order | 440 | 2.9% |
| o__unclassified_p__Basidiomycota | Order | 415 | 2.7% |
| o__Agaricales | Order | 322 | 2.1% |
| f__unclassified_p__Ascomycota | Family | 2736 | 18.1% |
| f__unclassified_k__Fungi | Family | 2591 | 17.2% |
| f__unclassified_o__Capnodiales | Family | 1197 | 7.9% |
| f__Teratosphaeriaceae | Family | 649 | 4.3% |
| f__unclassified_c__Sordariomycetes | Family | 490 | 3.2% |
| f__unclassified_p__Basidiomycota | Family | 415 | 2.7% |
| g__unclassified_p__Ascomycota | Genus | 2736 | 18.1% |
| g__unclassified_k__Fungi | Genus | 2591 | 17.2% |
| g__unclassified_o__Capnodiales | Genus | 1197 | 7.9% |
| g__unclassified_c__Sordariomycetes | Genus | 490 | 3.2% |
| g__unclassified_p__Basidiomycota | Genus | 415 | 2.7% |
| g__unclassified_f__Teratosphaeriaceae | Genus | 396 | 2.6% |
| s_unclassified_p_Ascomycota | Species | 2743 | 18.2% |
| s__unclassified_k__Fungi | Species | 2582 | 17.1% |
| s__unclassified_o__Capnodiales | Species | 1218 | 8.1% |
| s__unclassified_c__Sordariomycetes | Species | 493 | 3.3% |
| s__unclassified_p__Basidiomycota | Species | 418 | 2.8% |
| s__unclassified_f__Teratosphaeriaceae | Species | 401 | 2.7% |
|  |  |  |  |

**References**

1. Wei, Y., Lan, G., Wu, Z., Chen, B., Quan, F., Li, M., Su, S., Du, H. (2022). Phyllosphere fungal communities of rubber trees exhibited biogeographical patterns, but not bacteria. Environmental Microbiology, 24(8), 3777-3790. https://doi.org/10.1111/1462-2920.15894
2. Chen, J., Xu, H., He, D., Li, Y., Luo, T., Yang, H., Lin, M. (2019). Historical logging alters soil fungal community composition and network in a tropical rainforest. Forest Ecology and Management, 433, 228-239. https://doi.org/10.1016/j.foreco.2018.11.005
